# Laser Surface Texturing and Electropolishing of CoCr and Ti6Al4V-ELI Alloys for Biomedical Applications

**DOI:** 10.3390/ma13225203

**Published:** 2020-11-17

**Authors:** Jesús A. Sandoval-Robles, Ciro A. Rodríguez, Erika García-López

**Affiliations:** 1 Tecnologico de Monterrey, Escuela de Ingeniería y Ciencias, Ave. Eugenio Garza Sada 2501, Monterrey, Nuevo León 64849, Mexico; jesus.sandoval@tec.mx (J.A.S.-R.); ciro.rodriguez@tec.mx (C.A.R.); 2 Laboratorio Nacional de Manufactura Aditiva y Digital (MADiT)Apodaca, Nuevo León 66629, Mexico

**Keywords:** CoCr, Ti6Al4V ELI, laser texturing, electropolishing

## Abstract

The interplay between a prosthetic and tissue represents an important factor for the fixation of orthopedic implants. Laser texturing tests and electropolishing were performed on two materials used in the fabrication of medical devices, i.e., CoCr and Ti6Al4V-ELI alloys. The material surface was textured with a diode-pumped solid state (DPSS) laser and its effect on the surface quality and material modification, under different combinations of laser power and marking speed, were investigated. Our results indicate that an increment of energy per unit length causes an incremental trend in surface roughness parameters. Additionally, phase transformation on the surface of both alloys was achieved. Chemical analysis by energy dispersive X-ray spectrometer (EDX) shows the formation of (Co(Cr,Mo)) phase and the M_23_C_6_ precipitate on the CoCr surface; while quantitative analysis of the X-ray diffractometer (XRD) results demonstrates the oxidation of the Ti alloy with the formation of Ti_2_O and Ti_6_O from the reduction of the α-Ti phase. The behaviors were both related with an increase of the energy per unit length. Control of the final surface roughness was achieved by an electropolishing post-treatment, minimizing the as-treated values. After polishing, a reduction of surface roughness parameters was obtained in a range between 3% and 44%, while no changes in chemical composition or present phases were observed.

## 1. Introduction

CoCr and titanium alloys have been studied for many years in different biomedical areas such as cardiology, orthopedics, and dentistry, due to their biocompatibility, durability, corrosion resistance, high mechanical strength, high fatigue strength, and wear resistant properties [1]. The main applications for these materials are joint replacements [2], vascular stents [3], spinal disk replacements [4], and dental bridgework [5], among others. In particular, orthopedic implants require connectivity in the interphase between live bone and the implant surface. Therefore, the study of the implanted material is of great importance to accomplish a balance between the strength and the stiffness required for fixation of an implant to bone [6]. Additionally, the implant surface has evolved from solid smooth to a roughened surface and, recently, to porous in order to promote bone apposition and osseointegration [7]. In orthopedic implants, the main manufacturing techniques for engraving the surface of the material are laser ablation processing [8], plasma spraying [9], and sandblasting [10]. Laser-based processing has gained interest for texturing medical devices due to its high precision, high resolution, and suitability for selective changes in implant surfaces [11]. According to Cunha et al., using a direct writing method and a femtosecond laser radiation promotes laser-induced periodic surface structures, nanopillars, and microcolumns to improve implant osseointegration through the formation of bimodal roughness distribution [12]. In addition, laser texturing recreates a similar surface roughness as compared with acid etching which reproduces the texture of the bone matrix enhancing osteogenic cells to form new bone [13]. Some authors have studied the geometrical features involved in laser texturing and concluded that micro-groove or micro-dimple structures are the best option to improve engineering materials’ load capacity [14]. Götz et al. explained that the optimal pore size diameter was around 200 μm in laser textured implants, which was associated with the best cell adherence and the highest percentage of bone-implant contact which resulted in improvements on osseointegration [8]. Çelen et al. proposed that isotropic patterns promoted more controllable interfacial mechanical response and cleanliness of surfaces [5]. Additionally, surface treatment on implants influences the cellular behavior promoting cell attachment, considering factors, such as their chemical composition, roughness, and microtopography [14]. For example, Mirhosseini et al. explained that a high surface roughness could increase the number of cells that adhere to the substrate and, consequently the cell activity [15]. According to Soboyejo et al., smooth and alumina-blasted surfaces promoted random cell orientations which caused scar tissue, while micro-groove geometries promoted contact guidance to cells giving a narrow range of orientations [16]. Additionally, Nevins et al. demonstrated that laser ablated micro-grooved implant surface with depths and widths in a range between 8 and 12 µm placed on the collar of dental implants allowed bone and connective tissue attachment [17].

Table 1 presents a review of laser texturing of CoCr and titanium alloys including the process parameters and type of study performed. Electropolishing is used as an alternative method for surface engraving in orthopedic applications [18]. Szmukler-Moncler et al. studied acid treated titanium surfaces, and their results indicated that acid etching did not create a standard topography which was caused by controlling several parameters (i.e., acid mixture composition, temperature, treatment time, and prior treatment) [19]. Chen et al. reported a random surface with embedded Al_2_O_3_ particles and a relatively rough surface in blasting process, while laser ablated micro-grooved surfaces presented uniform surface morphologies with splatter patterns of resolidified material within and around the micro-grooved regions [20]. The processing chain, involving laser texturing and electropolishing, is of great interest in the medical field, where a perspective of mesotopography and microtopography take relevance. In the mesoscale, surface texturing of patterns reduces contact area to force to span the distance between structures and, consequently, bacteria growth [21], while microtopography allows cell proliferation and bone growth. The aim of this study was to evaluate CoCr and Ti6Al4V-ELI alloys processed through laser marking and electropolishing methods. Samples were characterized to study the influence of laser texturing parameters on surface topography and chemical composition.

## 2. Materials and Methods

### 2.1. Sample Preparation of CoCr and Ti6Al4V-ELI Alloys

Samples based on micro-melt Biodur CCM (Co-28Cr-6Mo) unannealed alloy (Carpenter, PA, USA) by specification of ASTM F1537 and Ti6Al4V ELI alloy (M. Vincent & associates, Bloomington, MN, USA) were laser textured and electropolished. Cylindrical samples of both materials with a diameter and length of 12.7 mm and 25.4 mm, respectively, were ground. A top arithmetical mean surface roughness (*R_a_*) of 0.4 µm and a ten-point surface roughness (*R_z_*) of 4 μm were obtained on the top surface. Samples were cleaned using a 70% isopropyl alcohol and 30% of DI water solution inside an ultrasonic bath for 3 min. Table 2 presents the chemical composition of CoCr Biodur alloy and Ti6Al4V ELI supplied by Carpenter, PA, USA and TiFast, Narni, Italy, respectively.

### 2.2. Laser Surface Texturing Experiments

A diode-pumped solid state (DPSS) laser marking system (EV25DS, Telesis, Circleville, OH, USA) with a maximum average power output of 25 W and an emission wavelength of 1064 nm was used for surface texturing. The laser system was focused on the top surface using a focal length of 100 mm resulting in a measured spot size diameter of 45 μm and an image field of view of 45 × 45 mm.

Each cylinder was mounted on a laboratory jack with an adjustable height platform in order to align the sample at the focal distance. The cylinder was inserted into a three-dimensional (3D) printed base fixture and the focal position was obtained using the guide light on the top surface of the cylindrical sample.

Table 3 presents the marking parameters used in both materials in terms of the conditions programmed in the machine.

**Figure 1:**
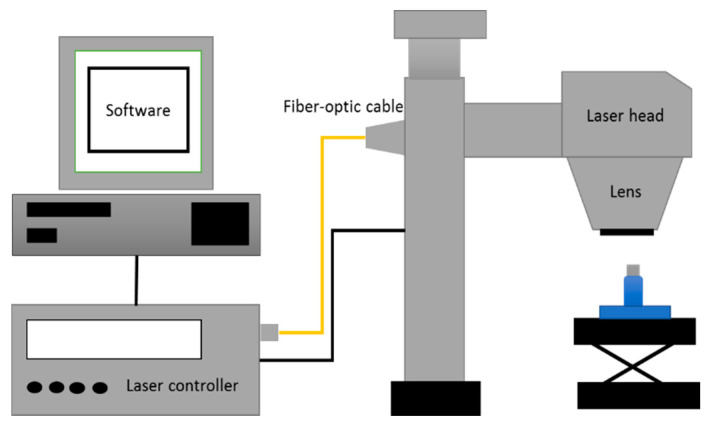
Laser texturing setup.

**Figure 2:**
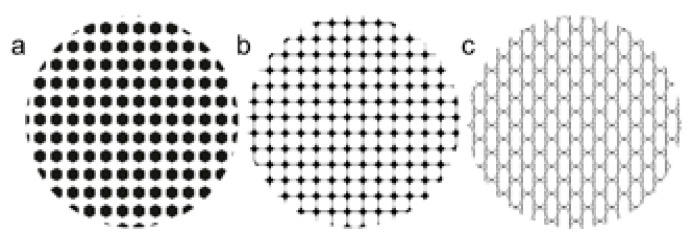
Surface designs. (**a**) Hexagonal; (**b**) Rhomboid; (**c**) Network.

Additionally, some laser parameters were fixed (i.e., laser frequency at 10 kHz, and pulse width at 5 μs). Energy per unit length (*E_l_*) was calculated as [27]:

(1) $$E_{l}=\frac{P}{v}$$

where *P* is the laser power and v is scanning speed. The experimental tests consisted of laser texturing a square area of 7 by 7 mm to determine the chemical composition, alloy phases, and surface roughness at different energy levels. The designs were programmed on a Telesis machine using Merlin software (Telesis, Circleville, OH, USA). In addition, three designs (i.e., hexagonal, network, and rhomboid) were drawn using Adobe Illustrator software (Adobe, San Jose, CA, USA), and then programmed as a bitmap image through the machine software. The designs and the square area were laser marked as individual pixels using the raster mode and a left to right path strategy. Figure 1 presents the experimental setup used for laser marking and Figure 2 illustrates the textured designs.

The laser marking parameters programmed for the three structures were a laser power of 21.25 W and a laser marking speed of 150 mm/s for both materials. These parameters were chosen according to our initial results and based on surface roughness measurements obtained after electropolishing.

### 2.3. Electropolishing Experiments

Samples were electropolished in an Esma E782-EP (EsmaInc, IL, USA) system. For the CoCr alloy, an Esma-brite E272 electrolyte (EsmaInc, IL, USA) solution was used as an etchant. It was based on ethylene glycol (<85 wt.%) and 2-hydroxyethyl hydrogen sulfate (<25 wt.%). The complete polishing process consisted of four stages (i.e., electropolish, neutralize, passivate, and clean the sample). The part was suspended using a metal clip tightened to a horizontal arm with a knurled screw, and it was fully submersed in the E272 electrolyte solution for 60 s at 60 °C, using 12 V of amplitude. Samples were neutralized using a NaHCO_3_ saturated solution, passivated with a 20% HNO_3_ and DI water solution for 2 min, and cleaned with a 70% isopropyl alcohol and DI water solution. For the Ti6Al4V ELI alloy, a solution with ethylene glycol (38 wt.%), HClO_4_ (10 wt.%), and DI water (52 wt.%) was employed as etchant. A beaker with an etchant solution was maintained inside a distilled water bath to avoid overheating, while the part was suspended with a metal clip inside the solution for 30 s, and the applied voltage was around 12.5 V relative to a carbon rod reference electrode. Samples were passivated and cleaned with the same solutions used for the CoCr alloy.

### 2.4. Surface Characterization

Chemical and surface characterization were performed after the samples were laser marked and electropolished. Surface roughness and 3D laser surface topography were measured with a confocal microscope (Axio CSM 700, Zeiss, Germany). All laser-textured samples were measured in three zones inside a square area. A scanning electron microscope (SEM) (EVO MA 25, Zeiss, Germany) coupled to an energy dispersive X-ray spectrometer (EDX) (Bruker, Harvard, MA, USA) was used to determine the surface chemical composition. Additionally, an X-ray diffraction study was performed to identify possible phase shifting at different energy levels. The diffractometer equipment (Empyrean, Malvern Panalytical, Malvern, UK) was adapted with an αCo radiation (Kα_1_ = 1.789 Å) for the Co-Cr alloy and αCu radiation (Kα = 1.54 Å) for the Ti alloy. Phase analysis was applied to all samples after the laser texturing to determine the parameters influence on the phases presented on the material. Quantification analysis for the phases present in the Ti alloy was obtained by Rietveld refinements using the MAUD software [28]. Phases considered are listed in Table 4.

## 3. Results

### 3.1. Laser Texturing and Electropolishing Tests

**Figure 3:**
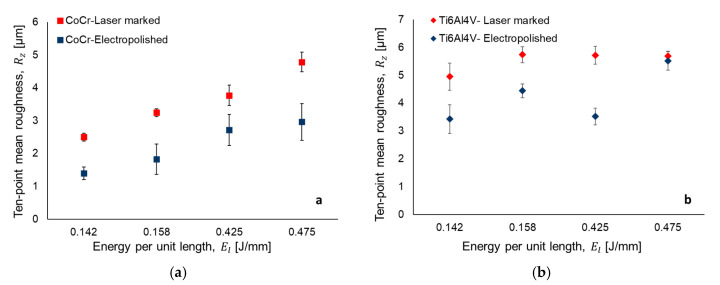
Ten-point mean roughness after laser texturing and electropolishing for (**a**) CoCr and (**b**) Ti6Al4V.

**Figure 4:**
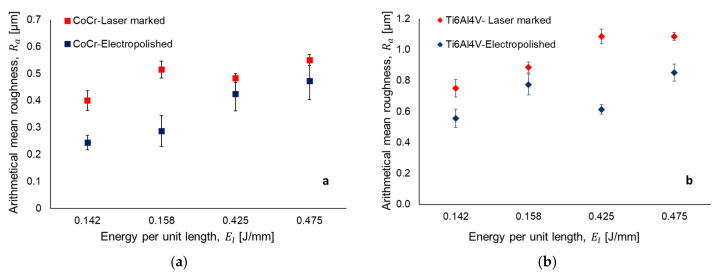
Arithmetical mean roughness after laser surface texturing and electropolishing for (**a**) CoCr and (**b**) Ti6Al4V.

Figure 3 and Figure 4 illustrate the results for the ten point mean and arithmetical mean surface roughness, respectively. The error bar represents the standard deviation in the measurement.

**Figure 5:**
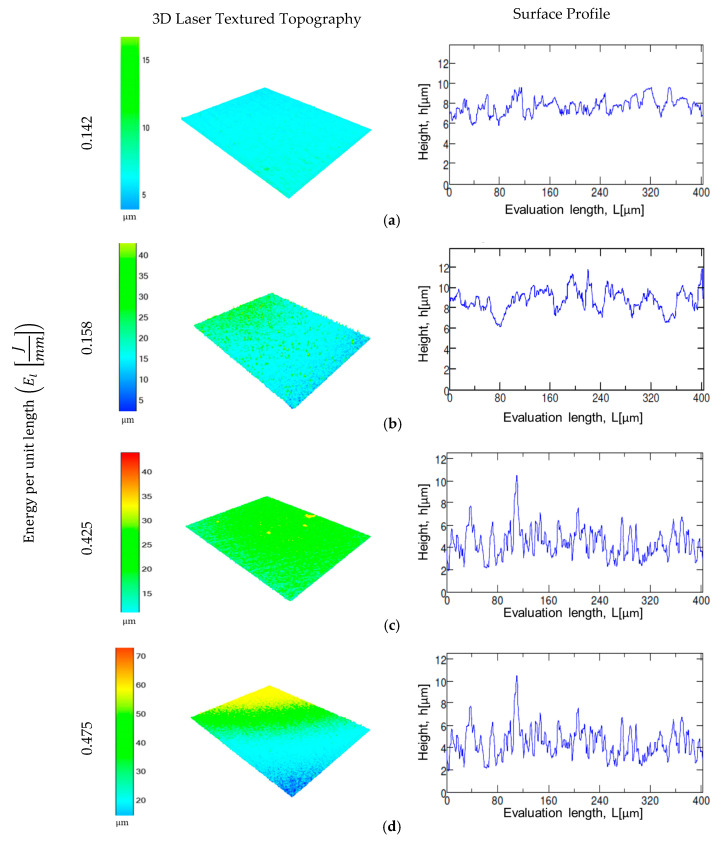
Laser surface topography of CoCr after being laser marked at different energy levels. (**a**) $E_{L}=0.142\;J/mm$; (**b**) $E_{L}=0.158\frac{J}{mm};\;\left(c\right)\;E_{L}=0.425\frac{J}{mm}$; (**d**)$\;E_{L}=0.475\;J/mm$.

**Figure 6:**
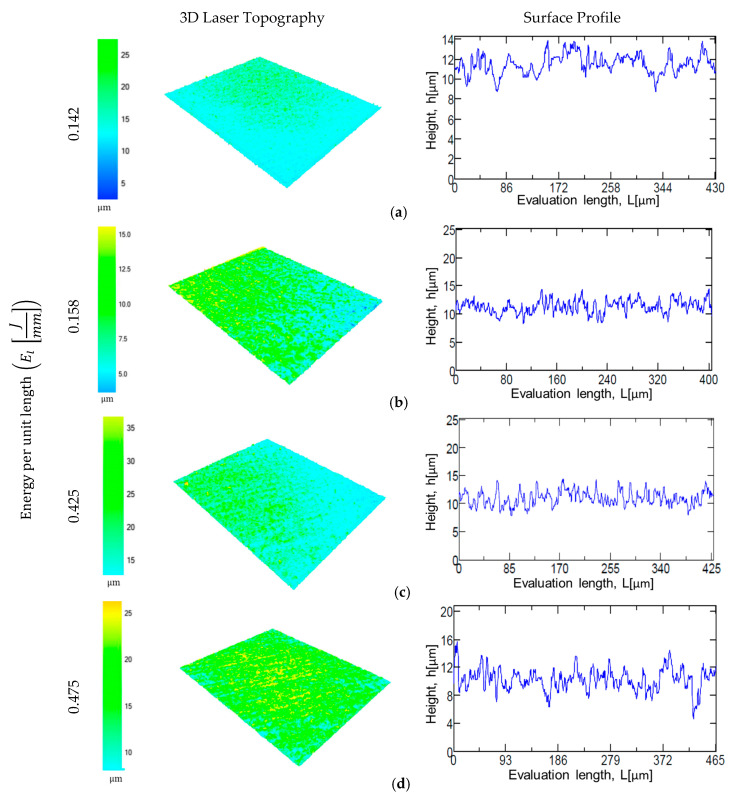
Laser surface topography of Ti6Al4V after being laser marked at different energy levels. (**a**) $E_{L}=0.142\;J/mm$; (**b**) $E_{L}=0.158\frac{J}{mm};\;\left(c\right)\;E_{L}=0.425\frac{J}{mm};\;$ (**d**) $E_{L}=0.475\;J/mm$.

The results indicate that, with the increase energy per unit length *E_l_* (J/mm), surface roughness values of the samples increase for both roughness parameters. This relation is explained by the combination of laser power and laser marking speed parameters using Equation (1). For example, a laser marking speed of 150 mm/s and laser power values of 21.25 W and 22.5 W (i.e., energy per unit length of 0.142 J/mm and 0.158 J/mm) represent the lower values of surface roughness in both materials as compared with 0.425 J/mm and 0.475 J/mm (Figure 3a,b). Figure 5 and Figure 6 present the 3D laser textured topography and the surface profile of CorCr and Ti6Al4V alloys. The images are classified based on the energy per unit length applied. Surface profile indicates the height achieved when the material is texturized. All samples were electropolished with the same treatment. Our results indicate a decrease in both surface roughness parameters. For CoCr alloy, a surface roughness reduction was found for Rz (i.e., 38% to 44%) and Ra parameters (i.e., 12% to 44%) (Figure 3a or Figure 4a). For the Ti6Al4V alloy, Rz parameter was reduced in a range between 3% and 38% (Figure 3b) and Ra parameter was reduced from 12% to 43% (Figure 4b).

**Figure 7:**
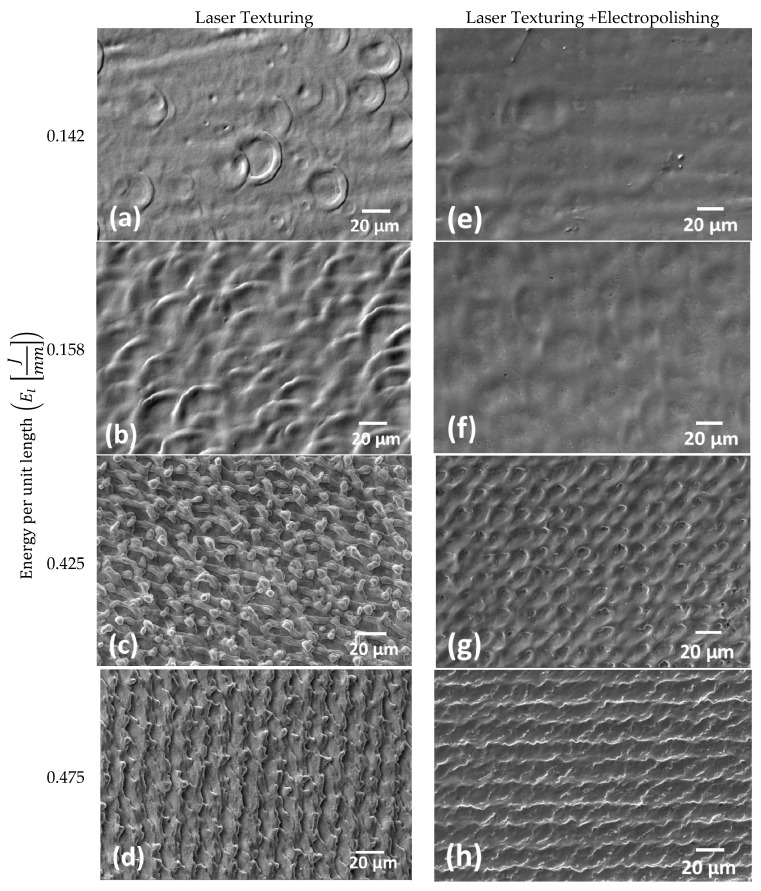
Laser texturing micrographs of CoCr surface quality after being laser marked. (**a**) $E_{L}=0.142\;J/mm$; (**b**) $E_{L}=0.158\frac{J}{mm};\;\left(c\right)\;E_{L}=0.425\frac{J}{mm};\;$ (**d**) $E_{L}=0.475\;J/mm$; and electropolished (**e**–**h**), at the same level of energy per unit length applied.

Figure 7 illustrates a qualitative study of the CoCr alloy surface observed in the SEM at 500× for laser marked (Figure 7a–d) and electropolished (Figure 7e–h) samples. Samples marked with the highest values of energy per unit length (Figure 7c,d) showed an under polished surface (Figure 7g,h). Additionally, samples with an energy per unit length between 0.142 J/ mm and 0.158 J/mm resulted with a well-polished surface.

**Figure 8:**
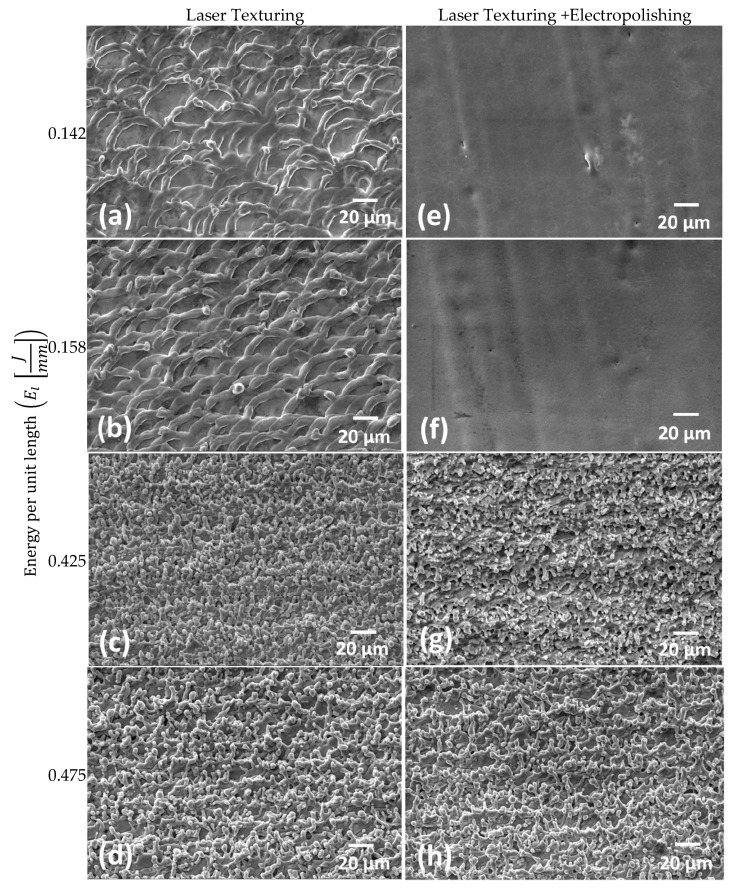
Laser texturing micrographs of Ti6Al4V ELI surface quality after being laser marked. (**a**) $E_{L}=0.142\;J/mm$; (**b**) $E_{L}=0.158\frac{J}{mm};\;\left(c\right)\;E_{L}=0.425\frac{J}{mm};\;$ (**d**) $E_{L}=0.475\;J/mm$; and electropolished (**e**–**h**), at the same level of energy per unit length applied.

Figure 8 presents a qualitative study of the Ti6Al4V ELI alloy for laser textured samples (Figure 8a–d) and electropolished samples (Figure 8e–h). Samples (Figure 8e,f) resulted efficiently polished while (Figure 8g,h) were not fully cleaned through polishing.

**Figure 9:**
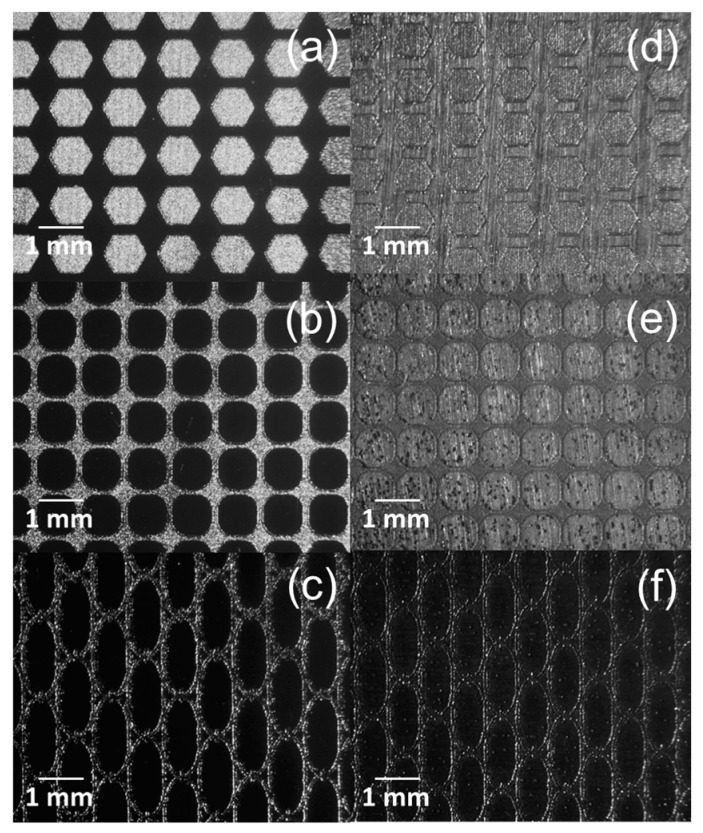
Laser texturing micrographs of CoCr alloy (**a**–**c**) and Ti6Al4V ELI (**d**–**f**) patterns; hexagonal, rhomboid, and network.

To complete the objective of this work, electropolishing time was based on the sample with less surface roughness in order to homogenize the exposure time of the samples and to avoid pitting corrosion defects. Figure 9 presents the SEM micrographs of hexagonal (Figure 9a,d), rhomboid (Figure 9b,e)and network design (Figure 9c,f) manufactured in CoCr and Ti6AL4V alloy using a magnification at 40×. The designed surfaces were laser textured using an energy per unit length of 0.142 J/mm and the same treatment used for samples electropolished in our previous results.

### 3.2. Chemical Composition

**Figure 10:**
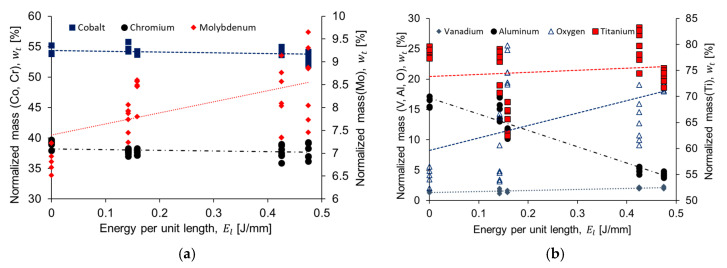
Chemical composition performed after laser texturing on (**a**) CoCr alloy and (**b**)Ti6Al4V alloy samples.

**Figure 11:**
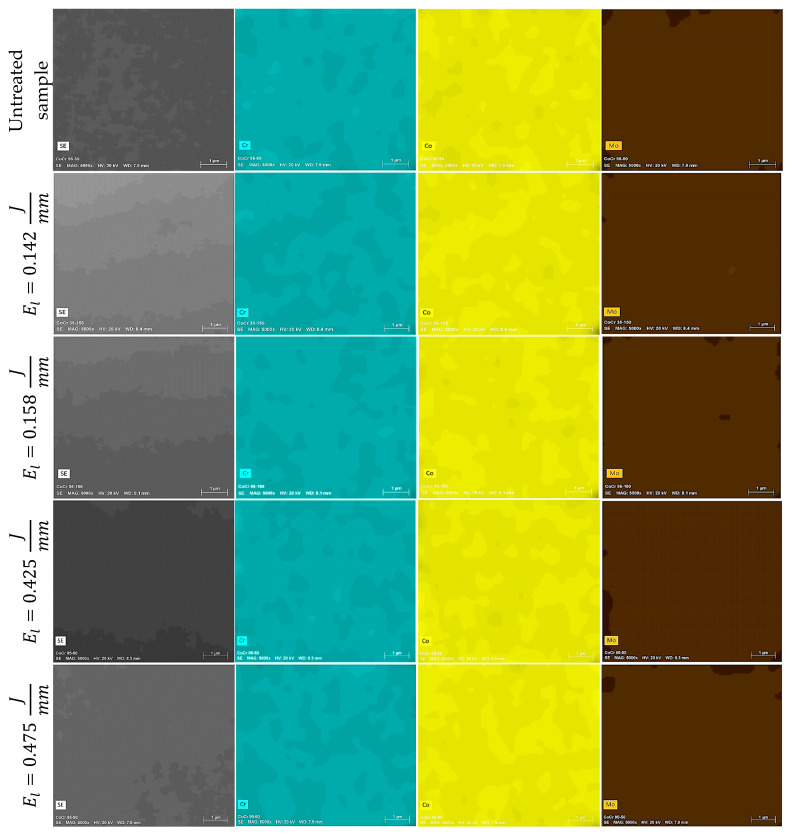
SEM-EDS maps (5000×) showing chemical elements found in the cross-section of laser textured CoCr samples using different energy per unit length. From left to right, the pictures correspond to topography (SE), chromium (Cr), colbalt (Co), molybdenum (Mo).

**Figure 12:**
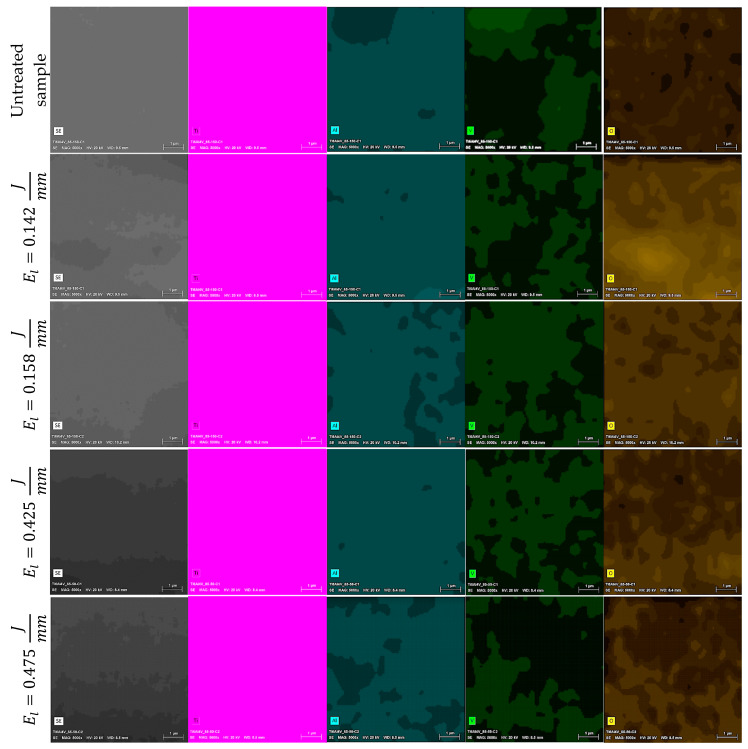
SEM-EDS maps (5000×) showing chemical elements found in the cross-section of laser textured Ti6Al4V samples using different energy per unit length levels. From left to right, the pictures correspond to topography (SE), titanium (Ti), aluminum (Al), vanadium (V), oxygen (O).

EDX analysis was performed to identify modifications of the chemical composition caused by increasing the applied laser energy. Figure 10a illustrates the chemical composition results for the laser marked CoCr alloy samples. Our results indicate a reduction of Co and Cr, while Mo increases with the increment of energy per unit length applied to the material. Figure 10b presents the chemical composition results for the Ti6Al4V ELI laser marked samples. From our results, there is a clear reduction of the aluminum mass percentage when energy per unit length is increased. These differences for chemical composition in both alloys were corroborated with ANOVA analysis (Table 5) for the different levels of energy per unit length (i.e., 0, 0.142, 0.158, 0.425, and 0.475 J/mm). Chemical composition fluctuation can be explained by variation in energy per unit length applied. In fact, this observation was corroborated by the correlation coefficient between energy per unit length and the chemical elements of CoCr alloy (Co, r = −0.338; Cr, r = −0.221; and Mo, r = 0.562) and Ti6Al4V (Ti, r = 0.093; Al, r = −0.949; V, r = 0.801; and O, r = 0.543). In addition to the chemical analysis on the textured surface, the cross-section of cylinders was investigated using EDS maps for CoCr and Ti6Al4V alloys (Figure 11 and Figure 12, respectively). The sets of images both show a qualitative distribution of the main elements present on each alloy. The variation of such elements is related to the level of energy applied. No contamination was observed in samples. However, further studies should be performed increasing the number of laser passes, which would have an influence on the formation of other compounds.

### 3.3. X-ray Diffraction

**Figure 13:**
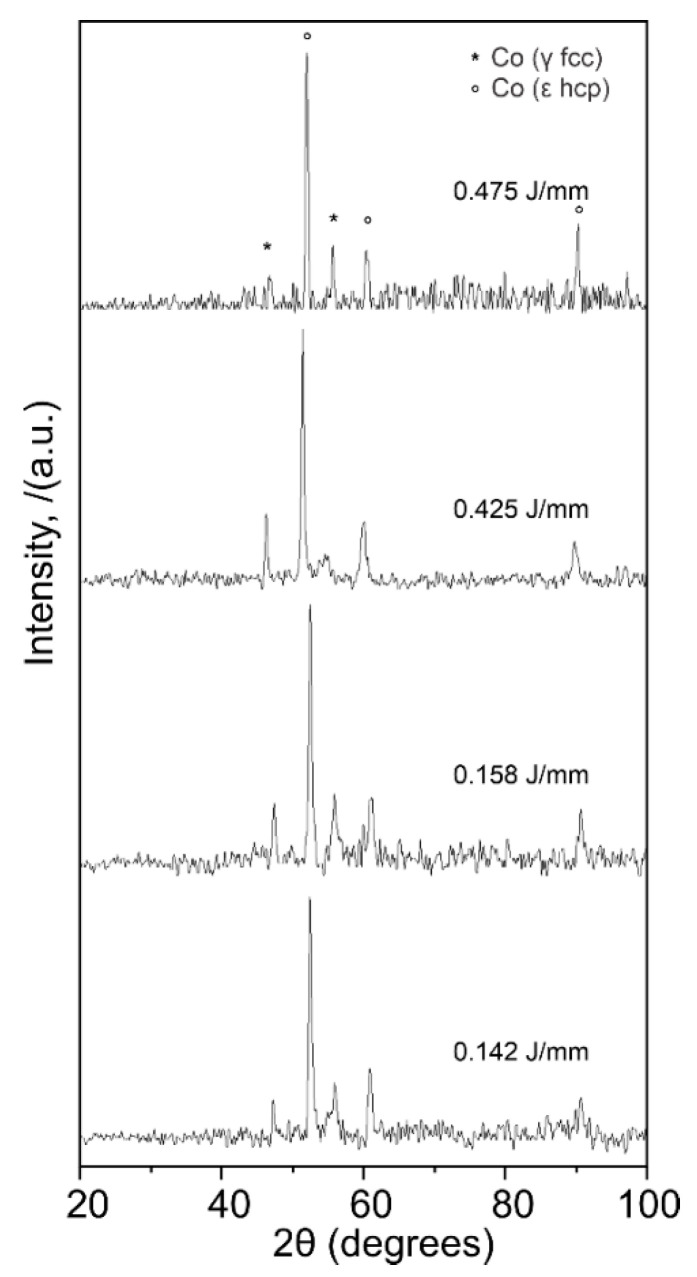
XRD patterns of the CoCr alloy samples after each laser textured sample. All phases correspond to Co-based phases.

**Figure 14:**
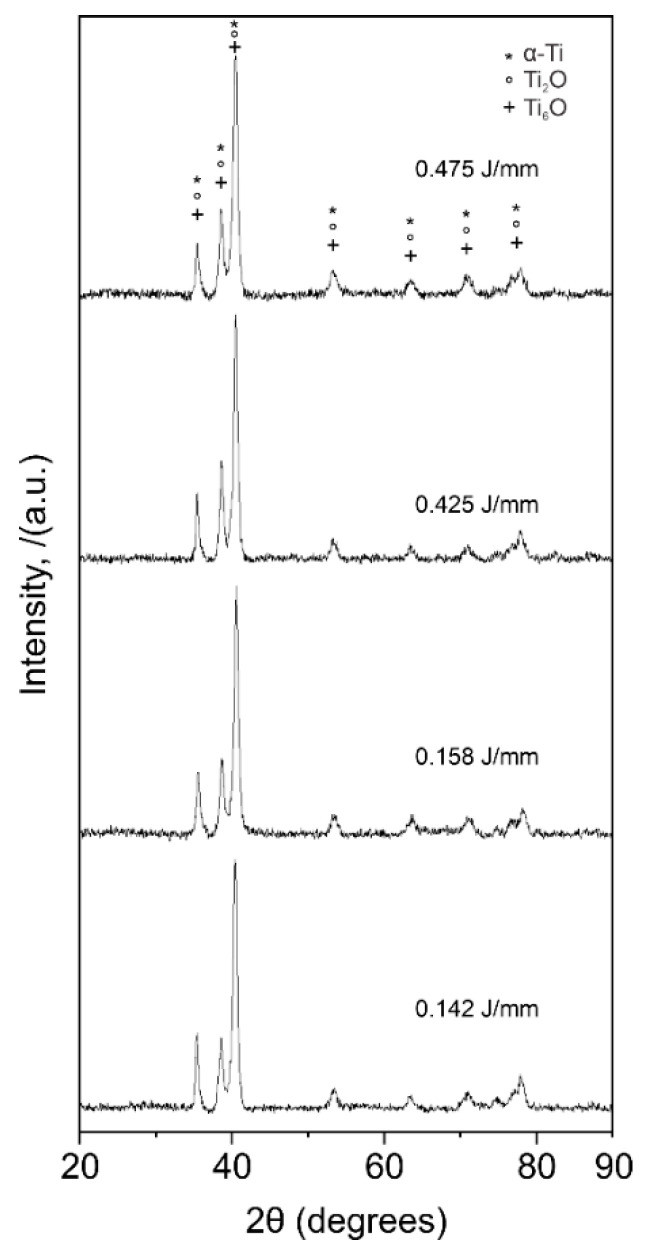
XRD patterns of the Ti alloy samples after each laser textured sample. All phases correspond to α-Ti and its low oxidized phases.

The X-ray diffraction analysis for the CoCr alloy shows the phases present on the samples after the laser treatment. These phases were identified as the Co-based, γ-fcc (ICDD card no. 14-238), and the ε-hcp (ICDD card no. 59-722) (Figure 13), which correspond to the Co-Cr 27% phase diagram. Formation of molybdenum carbides (M_23_C_6_) and σ phase (Co(Cr,Mo)) is expected, nevertheless, diffraction peaks for such precipitates were not visible as the (1010) of the ε-hcp (peak around 46° in 2θ) and the (111) of the γ-fcc (peak around 52° in 2θ) mask the main reflections of such phases. Several phases were identified in the Ti alloy (Figure 14), these phases were identified as α-Ti (ICDD card no. 11-198), Ti_2_O (ICDD no. 44-583), and Ti_6_O (ICDD no. 31-118). The formation of such phases is possible, according to the chemical composition of the alloy, but is expected to find differences in the formation of the mentioned phases due to the laser process. β phase was not observed in the diffraction analysis of the modified surfaces. Laser-based processes generate immediate melting and solidification of the sample. These high cooling rates promote the β phase transformation into α’ (martensitic) and α phases.

**Figure 15:**
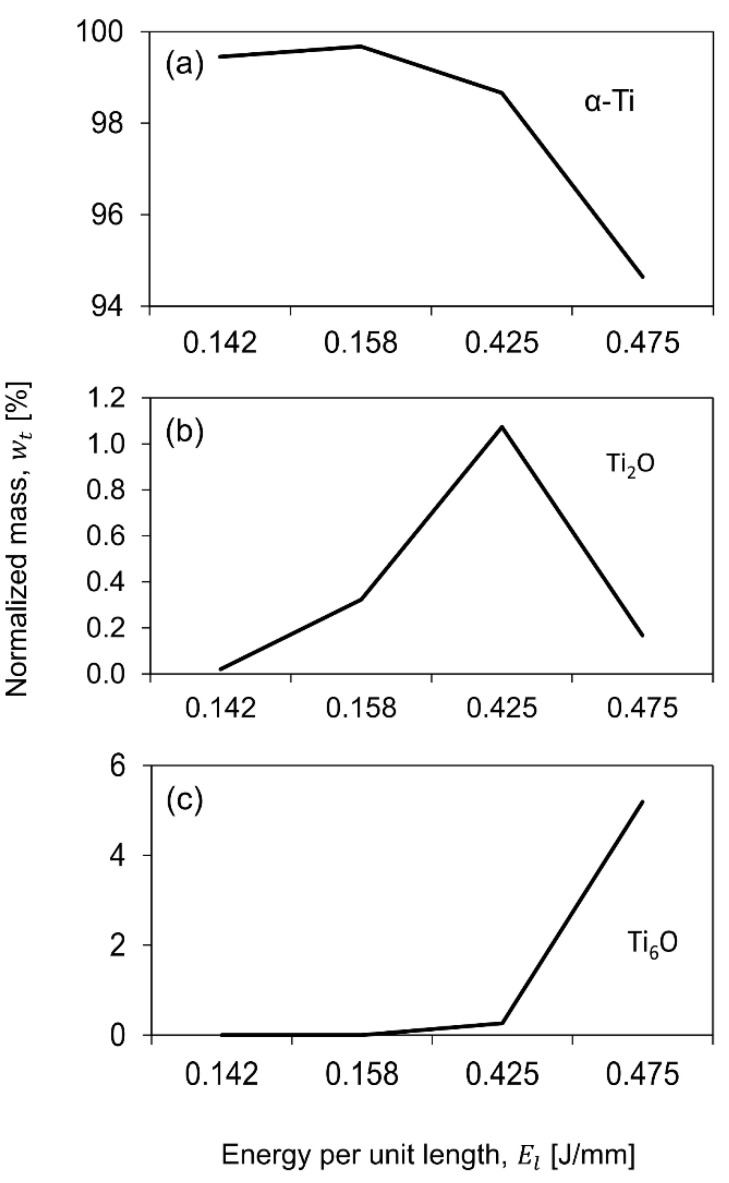
Quantitative analysis of the phases found in the laser treated Ti samples: normalized mass found for (**a**) α-Ti, (**b**) Ti_2_O and (**c**) Ti_6_O at different E_t_.

Several authors have reported no presence of the β phase in the heat-affected zones (HAZ) of laser-treated Ti6Al4V, which is also an effective strengthening method for this alloy [29,30,31]. The quantitative analysis of such phases was performed by the Rietveld method using the MAUD software. Results of this analysis are presented in Figure 15a, the α-Ti phase diminishes as the energy supplied to the surface increases, while in the same energy direction, an increment on the oxidized Ti phases (Figure 15b,c), is evident.

## 4. Discussion

Surface topography of textured zones in medical implants are of great interest given the interaction between the prosthesis and tissue. According to Ravi et al., a good integration of the implant to bone interface is a prerequisite for long-term implants stability [32]. According to Leone et al., the best visibility in samples treated with laser marking process implies low frequencies, low power, and high scanning speeds [33]. In our experiments, the interaction among these three parameters were not tested. However, low values of laser power and high values of scanning speed resulted in lower surface roughness values at a microscale (Figure 3 and Figure 4). Additionally, visibility of the marked zone can be measured as a contrast index (C) which increases with surface roughness due to the relation between the average gray values of marked area and the virgin surface [33]. In our study, an increment of energy per unit length (i.e., a marking speed of 50 mm/s) promoted heat-affected zones (HAZ) which caused an increase in the surface roughness parameter and, consequently, contrast index. According to Lavvafi et al., HAZ thickness increases by increasing the laser power during laser processing [34]. In addition, laser texturing parameters can modify material properties causing an effect in biocompatibility. For example, laser surface treatment can adjust the titanium oxide layer, which has a sterilization effect enhancing biocompatibility [11]. Boyd et al., explained that increasing the surface roughness of stainless steel promoted an increase in bacterial adhesion [35]. However, a rougher implant surface has effective bone to implant contact [36]. The laser marking of micro cavities in implants modifies the surface topography at a mesoscale. These 3D patterns have the advantage of minimizing stress shielding effect between the bone and an implant [5]. However, at the microscale, well-formed cavities have a surface roughness which depends on laser parameters. In this study, surface roughness was quantified on the bottom of the marked samples. Electropolishing has been demonstrated to be significantly improve corrosion resistance and increase biocompatibility and homogeneity [37]. To the best of our knowledge, there are no studies explaining the effect of electropolished samples after being laser marked. However, there are many experimental works that have studied the response of surfaces being treated with electropolishing after laser cutting [38,39], laser sintering [40] or have used electropolishing as an alternative method to engrave surfaces as laser technologies [41]. Further studies should be performed to study the different surface topographies and their biological response.

In terms of chemical composition of the Ti6Al4V alloy, there is a correlation between increasing energy per unit length and an increase in the oxygen level, as shown in Figure 10b. According to Leone et al., surface roughness and oxidation are both important in determining contrast levels [33]. S. Spriano et al. reported on an EDX analysis of a treated Biodur CoCr alloy and observed that the elements of Co and Ta registered a drastic decrease and increase, respectively, in correspondence to a higher temperature (1000 °C) caused by the applied thermal treatment [42]. From the EDX results, the CoCr alloy investigated in this work has no presence of a tantalum element in the chemical composition (Figure 10a). However, our results indicate a reduction of Co and Cr, while Mo increases with an increase of energy per unit length applied to the material. According to Mineta et al., a combination of low carbon and high molybdenum content in Co-Cr alloys is more suitable to form the intermetallic σ phase (Co(Cr,Mo)) or the M_23_C_6_ precipitate at low temperatures and high cooling rates, which both can experience due to laser interaction on the surface [43]. This can be related to the changes in the chemical composition observed in the EDX results. The formation of the intermetallic Mo phases and precipitates could not be verified by the XRD analysis. As mentioned before, all the identified phases, and expected ones, as a result of the surface treatment, have the same crystallographic reflections. In order to fully identify the formed precipitates during the process, precipitates need to be fully separated from the alloy matrix. For Ti6Al4V alloy (Figure 10b), there is a clear reduction of the aluminum mass percentage when energy per unit length is increased. Al and V behavior is expected due to the alloy composition and the process parameters involved. A minor increase of the V content may be due to a high cooling rate; while the Al content diminishes as the energy increases, as the V inhibits the formation of Al_2_O_3_, hence the evaporation of the aluminum during the surface treatment [44]. A similar trend was found by Juetcher et al. According to their results, when energy input increased and, consequently, the top surface temperature, there was a loss of light elements such as aluminum due to the increment of scanning speed [45]. Additionally, the formation of titanium oxides was possible as a result of the laser processing, and the XRD patterns indexation was coherent with the EDX results, as there were no signs of the titanium β-phase, or the high titanium oxidized phase (TiO_2_); while Ti_2_O and Ti_6_O were formed by oxygen diffusion in the titanium α-phase [46].

## 5. Conclusions

This study reports the use of a process chain, i.e., laser texturing and electropolishing, in order to treat surfaces on CoCr and TI6Al4V alloys, intended for medical implants. The effect of this process chain on surface topography and surface chemical composition was investigated. The conclusions are summarized as follows:

- The surface topography modification was related to the HAZ by means of the energy per unit length during laser texturing. For CoCr alloy, an increase in the energy per unit length (*E_l_*) shows a linear increment of surface roughness parameters, whilst for the Ti6AL4V-ELI alloy, the laser effect at high *E_l_* remained constant.
- The electropolishing test showed that the final surface roughness could be controlled to comply with different medical applications.
- The laser texturing test showed a surface modification in terms of the chemical composition, and hence the phases present on the treated surface.
- The chemical analysis of the CoCr alloys provided indications of the formation of intermetallic σ phase (Co(Cr,Mo)) or the M_23_C_6_ precipitate. More studies are needed to fully characterize this precipitates morphology as they are related with wear and corrosion resistant in implants.
- A reduction in the α-Ti and an increase in the formation of Ti oxides (which have a sterilization effect enhancing biocompatibility) was observed with increasing energy per unit length. This was also achieved due to the chemical composition of the Ti6AL4V-ELI alloy and the high heating/cooling rate of the laser process.

## Figures and Tables

**Table 1:** Literature review of laser texturing.

Ref.	Year	Material	Geometry Texturized	Laser Parameters		Results			
						Chemical Composition	Geometrical/Surface Quality	Cell Attachment	Mechanical Properties/Tribology
[20]	2006	Ti6Al4V	Parallel grooves	Laser type	UV		X	X	
				Wavelength (nm)	355				
				Scanning speed (mm/s)	250				
				Pulse frequency (kHz)	50				
				Avg. power (W)	1.65				
				spot size (µm)	8.5				
[22]	2011	Titanium	Pits	Laser type	Yb fiber				X
				Wavelength (nm)	1060				
				Avg. power (W)	-				
				Scanning speed (mm/s)	50–150				
				P. frequency (kHz)	-				
[23]	2011	Ti6Al4V	Dots and lines	Laser type	Yb Fiber	X	X	X	
				Wavelength (nm)	1060				
				Avg. power (W)	8				
				Pulse duration (ns)	70				
				P. frequency (kHz)	20–200				
[10]	2012	Ti6Al4V	Dimples	Laser type	Nd-YAG				X
				Wavelength (nm)	1064				
				Laser avg. power (W)	10				
				P. frequency (kHz)	10				
				Scanning speed (mm/s)	5				
[24]	2013	Ti6Al4V	Periodic waves	Laser type	Yb:KWY	X			
				Wavelength (nm)	1030				
				Scanning speed (mm/s)	0.8				
				P. frequency (Hz)	50				
[25]	2013	Co-Cr-Mo	Square, triangle, and circle	Laser type	DPSS				X
				Wavelength (nm)	1064				
				Laser power (W)	50				
				P. frequency (kHz)	20				
				Spot size (µm)	15				
[14]	2014	Co-Cr-Mo	Square, triangle, and circle	Laser type			X	X	
				Wavelength (nm)	1064				
				Avg. power (W)	20				
				Scanning speed (mm/s)	50–200				
				P. frequency (kHz)	3–5.5				
[26]	2015	Co-Cr-Mo	Petaloid	Laser type	VAN		X		X
				Wavelength (nm)	532				
				Laser power (mW)	50				
				P. frequency (kHz)	100				
				Pulse duration (ps)	10				

**Table 2:** Chemical composition.

**Chemical Composition CoCr (wt.%)**							
Co	65.45	Cr	27.34	Mo	5.47	Mn	0.69
Si	0.67	N	0.16	Fe	0.07	Ni	0.06
W	0.02	O	0.02	Cu	0.01	Al	0.01
Nb	0.01	C	0.037	Ti	0.005	P	0.004
B	0.003	S	0.002				
**Chemical Composition Ti6Al4V-ELI (wt.%)**							
Al	5.95	C	0.015	Fe	0.10	H	0.003
N	0.007	O	0.104	V	4.03		

**Table 3:** Laser marking texturing parameters.

Laser Power, P (W)	Marking Speed, v (mm/s)	Energy per Unit Length (J/mm)
21.25	150	0.142
23.75	150	0.158
21.25	50	0.425
23.75	50	0.475

**Table 4:** Crystalline structures of identified phases in the laser-treated Ti alloy samples.

Phase	Crystalline System	Space Group	Cell Parameters (Å)
α-Ti	Hexagonal	P63/mmc	a = b= 2.936, c = 4.679
Ti_2_O	Trigonal	P-3m1	a = b = 3.465, c = 4.049
Ti_6_O	Trigonal	P31c	a = b = 5.14, c = 9.48

**Table 5:** Analysis of variance of CoCr according to energy per unit length levels.

Source	Mean ± St. dev.	Range	F-Value	P-Value
CoCr alloy				
Cobalt (Co)	54.043 ± 0.669	(51.980, 55.780)	3.16	0.029
Chromium (Cr)	37.940 ± 0.947	(35.890,39.690)	2.66	0.053
Molybdenum (Mo)	8.018 ± 0.773	(6.520, 9.650)	9.32	0.000
Ti6Al4V alloy				
Titanium (Ti)	75.015 ± 5.398	(62.46,83.29)	30.12	0.000
Aluminum (Al)	10.213 ± 5.214	(3.76, 17.16)	232.67	0.000
Vanadium (V)	1.7394 ± 0.3574	(1.10, 2.34)	18.81	0.000
Oxygen (O)	13.18 ± 7.43	(2.01, 25.54)	38.13	0.000
